# Robot‐assisted partial nephrectomy using the Hugo™ RAS System: first multicentre study and Tetrafecta achievement

**DOI:** 10.1111/bju.70009

**Published:** 2025-10-06

**Authors:** Francesco Prata, Paolo Dell'Oglio, Angelo Mottaran, Riccardo Bertolo, Stefano Tappero, Pietro Piazza, Francesca Montanaro, Alessandro Veccia, Alberto Ragusa, Alberto Caviglia, Francesco Tedesco, Angelo Civitella, Andrea Iannuzzi, Tommaso Saccucci, Giovanni Muto, Aldo Massimo Bocciardi, Eugenio Brunocilla, Roberto Mario Scarpa, Alessandro Antonelli, Riccardo Schiavina, Antonio Galfano, Rocco Papalia

**Affiliations:** ^1^ Department of Urology Fondazione Policlinico Universitario Campus Bio‐Medico Rome Italy; ^2^ Research Unit of Urology, Department of Medicine and Surgery Università Campus Bio‐Medico Rome Italy; ^3^ Department of Urology ASST Grande Ospedale Metropolitano Niguarda Milan Italy; ^4^ Division of Urology I.R.C.C.S. Azienda Ospedaliero‐Universitaria di Bologna Policlinico Sant'Orsola Bologna Italy; ^5^ Urology Unit, Department of Surgery, Dentistry, Pediatrics and Gynecology University of Verona, A.O.U.I Verona Italy; ^6^ Department of Urology GVM – Maria Pia Hospital Turin Italy

**Keywords:** docking, Hugo RAS system, renal neoplasm, nephron‐sparing surgery, robotic, partial nephrectomy, Tetrafecta

## Abstract

**Objective:**

To evaluate the perioperative outcomes and the rate of Tetrafecta achievement in robot‐assisted partial nephrectomy (RAPN) cases performed using the Hugo™ robot‐assisted surgery (RAS) system (Medtronic, Minneapolis, MN, USA).

**Patients and Methods:**

Data from five tertiary care referral centres performing RAPN with the Hugo RAS System since October 2022 were collected. For the study purpose, a novel Tetrafecta was defined as a proxy for surgical quality. This included the coexistence of negative surgical margins, absence of intraoperative and Clavien–Dindo Grade ≥II postoperative complications, and 90‐day decline in estimated glomerular filtration rate of no more than 30% from baseline.

**Results:**

A total of 140 patients, who underwent RAPN with the Hugo RAS system, were included. The median (interquartile range [IQR]) docking, console, and total operative times were 5 (4–7) min, 79 (45.5–135) min, and 150 (96–205) min, respectively. The median (IQR) estimated blood loss was 150 (100–300) mL. No additional port placements were required, and no conversions to open surgery occurred. Overall, postoperative complications were observed in 20.7% of cases, 5% of which were classified as major (Clavien–Dindo Grade >II). Tetrafecta was achieved in 80% of cases. Multivariate logistic regression analysis showed that R.E.N.A.L. (Radius, Exophytic/Endophytic, Nearness to collecting system or sinus, Anterior/Posterior, Location) nephrometry score (odds ratio [OR] 0.69; *P* = 0.01) and Hugo RAS surgical experience (OR 1.03; *P* = 0.02) were independent predictors of Tetrafecta achievement.

**Conclusions:**

Relying on the largest multicentre series, our findings demonstrate that this novel robotic platform enables safe and effective RAPN, achieving a satisfactory Tetrafecta rate. These results would support the adoption of the Hugo RAS system to perform RAPN.

**Abbreviations**
BMI: body mass index
cT: clinical T Stage
EAU: European Association of Urology
EAUiaiC: EAU Intraoperative Adverse Incident Classification
EBL: estimated blood loss
eGFR: estimated GFR
NSS: nephron‐sparing surgery
OR: odds ratio
(RA)PN: (robot‐assisted) partial nephrectomy
PSM: positive surgical margin
pT: pathological T Stage
RAS: robot‐assisted surgery
R.E.N.A.L.: Radius, Exophytic/Endophytic, Nearness to collecting system or sinus, Anterior/Posterior, Location
WIT: warm ischaemia time

## Introduction

Partial nephrectomy (PN) represents the preferred surgical option for treating localised RCC, whenever technically feasible [1, 2, 3]. This approach reduces the likelihood of chronic kidney disease while maintaining oncological safety [4]. For surgeons, the minimally invasive pure laparoscopic approach to PN is associated with challenges in terms of limited dexterity, ergonomics, and steep learning curves [5].

Robot‐assisted surgery (RAS) represented a step ahead in the field of minimally invasive surgery, particularly in urology [6]. While the da Vinci® Surgical System (Intuitive Surgical Inc., Sunnyvale, CA, USA) has dominated the robotic surgery market for two decades, the recent introduction of the Hugo™ RAS system (Medtronic, Minneapolis, MN, USA) has offered to urologists a new platform for performing different urological interventions, including PN [7, 8].

However, compared to the da Vinci, there is limited evidence of the Hugo RAS system's clinical application, particularly for PN. The surgical set‐up, feasibility, safety profile, and clinical outcomes of Hugo RAS system robot‐assisted PN (RAPN) are limited to small sample size single‐centre series, with relatively short follow‐up. Moreover, data on functional outcomes are lacking [7, 8, 9, 10, 11].

To fill this gap, we have put together the largest multicentre case series available to date of RAPN with the Hugo RAS system. We aim to assess feasibility, safety profile, and clinical effectiveness.

## Patients and Methods

### Study Design and Population

The current study is based on a prospectively‐maintained board‐approved database that collected data on patients with renal masses who underwent RAPN with the Hugo RAS system between October 2022 and August 2024 at five Italian tertiary care referral centres for urological RAS.

In two of these centres, where the Hugo RAS system was the only available robotic system, consecutive, non‐selected patients were enrolled. In other participating centres, with multiple platforms, patients were scheduled for surgery with the Hugo RAS system depending on platform availability in the operating room, without any specific restrictions.

All the surgical team members fulfilled the official Medtronic training program.

### Data Collection

Detailed baseline data together with pre‐, intra‐, and postoperative information were collected. Docking times were considered from the insertion of the last trocar to the validation of all tilt and docking angles for the robotic arms. Intraoperative complications were recorded and classified using the Intraoperative Adverse Incident Classification (EAUiaiC) as recommended by the European Association of Urology (EAU) ad hoc Complications Guideline panel [12]. We also fulfilled the quality standards for accurate and comprehensive reporting of intraoperative adverse events, as outlined by the Intraoperative Complications Assessment and Reporting with Universal Standards (ICARUS) Global Surgical Collaboration Project [13] (Table S1). To ensure standardised and transparent reporting, perioperative complications were classified according to the EAU Guidelines on the reporting and grading of surgical complications (Table S2) [14]. Postoperative complications were graded according to the Clavien–Dindo classification [15]. Surgical experience for each individual patient was defined as the cumulative number of RAPN interventions performed by each surgeon before the patient's procedure [16].

### Surgical Technique

All surgeons participating in the study had solid expertise with >400 PN procedures performed.

**Fig. 1:**
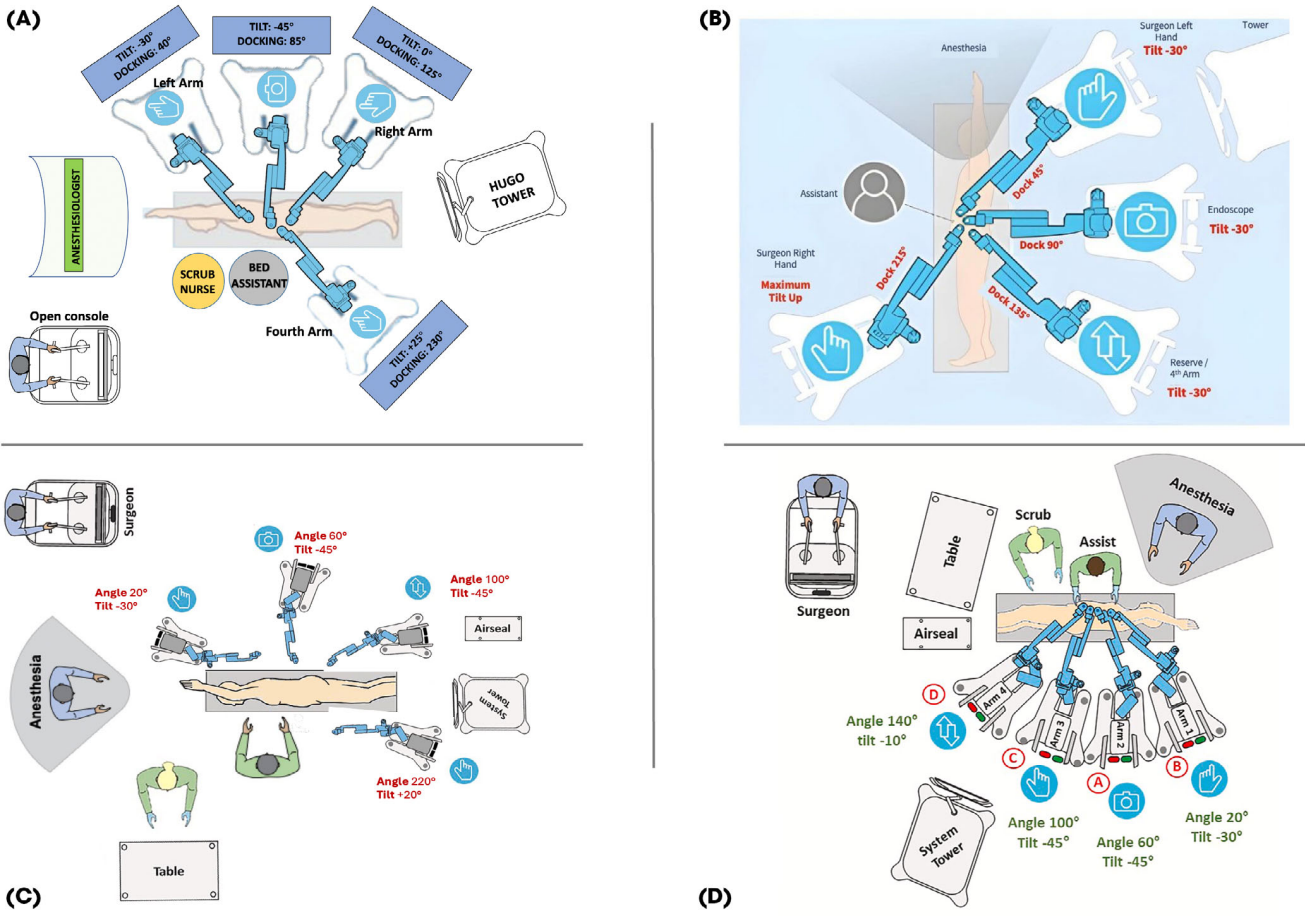
Hugo^TM^ RAS docking configurations at participating centers. **(A)** Configuration used at Institutions 1 and 5. **(B)** Configuration used at Institution 2. **(C)** Configuration used at Institution 3. **(D)** Configuration used at Institution 4.

The different surgical settings and trocar placement for the Hugo RAS system have been previously described [7, 8, 11] (detailed in Fig. 1).

The surgical technique for RAPN using the Hugo RAS system was chosen at the surgeon's discretion following the recently identified and approved steps by the Delphi consensus group [17]. No standardised protocol was enforced for pre‐, intra‐, or postoperative management. Common elements of the procedure included patient positioning in a modified flank position, docking of the robotic arms, and kidney dissection and tumour anatomical resection [18]. Renal hilum clamping was performed based on the surgeon's preference. Warm ischaemia time (WIT) was recorded in on‐clamp cases. Techniques for tumour localisation, resection, and renorrhaphy varied between institutions but followed the general principles of oncological safety and maximised kidney preservation. Either transperitoneal or retroperitoneal approaches were utilised, depending on patient's features, tumour location, and the surgeon's preference. According to EAU guidelines, the follow‐up protocol involved routine blood tests, monitoring of renal function, and CT scans every 3–6 months depending on the patient's risk group [19, 20].

### Study Endpoints and Statistical Analysis

First, the present study aimed to describe the perioperative outcomes of the largest multicentre series of RAPNs performed using the new Hugo RAS System, regardless of the technique employed.

Second, the present report aimed to define and assess a novel Tetrafecta as a proxy for surgical quality, defined by the simultaneous achievement of: absence of EAUiaiC and postoperative Clavien–Dindo Grade ≥II complications, negative surgical margins, and decline in estimated GFR (eGFR) of no more than 30% from baseline value at 90 postoperative days.

No formal sample size calculation was performed. This retrospective observational study was designed to provide a descriptive analysis of perioperative outcomes and to assess the feasibility and applicability of the Tetrafecta composite metric in a real‐world cohort of patients undergoing RAPN with the Hugo RAS system. Given the exploratory nature of the study and the absence of formal hypothesis testing, the entire consecutive sample available during the study period was included in the analysis.

Established recommendations for statistical analyses, reporting and interpretation of the results were applied [21]. Medians and interquartile ranges (IQRs) and frequencies and proportions were reported for continuous and categorical variables, respectively. A repeated‐measures ANOVA was performed to compare eGFR values measured at three timepoints: preoperatively, at hospital discharge, and at last follow‐up. This method accounts for the within‐subject correlation of repeated measurements. Uni‐ and multivariate logistic regression models were employed to evaluate the independent predictors of Tetrafecta achievement. To prevent overfitting and ensure model robustness, we included a predefined set of 10 covariates commonly reported in the literature as clinically relevant for RAPN outcomes. These included: age at surgery, body mass index (BMI), Charlson Comorbidity Index score, diabetes, blood hypertension, baseline eGFR, R.E.N.A.L. (Radius, Exophytic/Endophytic, Nearness to collecting system or sinus, Anterior/Posterior, Location) nephrometry score, ischaemia technique (on‐ vs off‐clamp), WIT, and surgeon Hugo RAS‐specific surgical experience. All variables were retained in the multivariate model regardless of their univariate significance, in line with methodological guidance aimed at minimising the risk of data‐driven selection [21]. Moreover, we ensured an events‐per‐variable ratio of at least 10 to preserve statistical power and prevent overfitting.

To explore the impact of surgical experience on perioperative outcomes, two additional analyses were performed. First, docking time was analysed as a continuous outcome variable across the progressive case number to assess for a potential learning effect. A linear regression model was used, with docking time (min) as the dependent variable and case number as the independent predictor. Second, to investigate the potential influence of surgical experience on perioperative complications, we treated the sequence of procedures as a continuous variable and used logistic regression to assess whether complication rates varied with increasing case number. All complications were considered, regardless of severity. Due to the low number of total events, this analysis was considered exploratory and primarily descriptive in nature. Statistical analyses were performed using STATA software (StataCorp. 2021, Release 17, College Station, TX, USA). All tests were two‐sided, with a significance level set at *P* < 0.05.

## Results

### Patient Characteristics

Overall, 140 patients treated with RAPN were included in the study (Table 1, Fig. S1). The median (IQR) tumour size was 32 (22–46) mm. Most treated masses were classified as clinical (c)T1a (101 [72.1%]), while T1b tumours represented 21.4% of the cases. Notably, nine patients (6.5%) had clinical (c)T2 disease. The median (IQR) R.E.N.A.L. nephrometry score was 6 (5–8).

**Table 1:** Baseline and demographic data of RAPN with the Hugo RAS system.

Variable	Value
Total number of RAPNs	140
Age, years, median (IQR)	63 (55.5–72)
Gender, *n* (%)	
Male	89 (63.6)
Female	51 (36.4)
BMI, kg/m^2^, median (IQR)	26.1 (24–29)
ASA score, *n* (%)	
I	4 (2.9)
II	100 (71.4)
III	36 (25.7)
IV	0 (0)
Charlson Comorbidity Index score, median (IQR)	4 (2–5)
Diabetes, *n* (%)	20 (14.3)
Hypertension, *n* (%)	71 (50.7)
Preoperative haemoglobin, g/L, median (IQR)	144 (134–153)
Preoperative creatinine, mmol/L, median (IQR)	0.0831 (0.0707–0.0981)
Preoperative eGFR, mL/min/1.73 m^2^, median (IQR)	79.6 (65–93.9)
Preoperative CKD Stage, *n* (%)	
1	43 (30.7)
2	73 (52.1)
3a	16 (11.4)
3b	6 (4.3)
4	2 (1.5)
5	0 (0)
Clinical tumour size, mm, median (IQR)	32 (22–46)
Clinical T Stage, *n* (%)	
T1a	101 (72.1)
T1b	30 (21.4)
T2a	7 (5)
T2b	2 (1.5)
Side, *n* (%)	
Right	79 (56.4)
Left	59 (42.1)
Bilateral	2 (1.5)
R.E.N.A.L. nephrometry score, median (IQR)	6 (5–8)
Cystic features, *n* (%)	22 (15.7)

CKD, chronic kidney disease.

### Perioperative Outcomes

The median (IQR) docking time was 5 (4–7) min. The median (IQR) total operative time was 149.5 (96–205) min, while the median (IQR) console time was 79 (45.5–135) min (Table 2). The median estimated blood loss (EBL) was 150 (100–300) mL. The median (IQR) enucleation time was 12 (8–20) min. In all, 50 patients (35.7%) were performed on‐clamp, with a median (IQR) WIT of 16 (12–21) min; selective clamping was applied in 3.6% of the whole cohort. No conversions to pure laparoscopic or open approach occurred. Intraoperative complications were registered in two patients (1.4%, transfusions). Postoperative complications occurred in 20.7% of patients (Table 2). Among these, major complications (Clavien–Dindo Grade IIIa) were observed in 5% of cases. Specifically, we recorded three cases of renal pseudoaneurysm that required selective arterial embolisation, two cases of pneumothorax managed with chest tube placement, one case of urinary leakage treated with the insertion of a JJ ureteric stent, and one case of symptomatic lymphocele that required percutaneous drainage. The median (IQR) length of stay was 3 (3–4) days. The overall 30‐day readmission rate was 2.9%, which was exclusively related to the management of the aforementioned complications. No perioperative mortalities were reported.

**Table 2:** Perioperative data, short‐term oncological and functional outcomes of RAPN (*n* = 140) with the Hugo RAS system.

Variable	Value
Docking time, min, median (IQR)	5 (4–7)
Console time, min, median (IQR)	79 (45.5–135)
Operative time, min, median (IQR)	149.5 (96–205)
Enucleation time, min, median (IQR)	12 (8–20)
WIT, min, median (IQR)	16 (12–21)
Hilum approach, *n* (%)	
Selective clamp	5 (3.6)
On‐clamp	45 (32.1)
Off‐clamp	90 (64.3)
Surgical route, *n* (%)	
Transperitoneal	136 (97.1)
Retroperitoneal	4 (2.9)
Renorrhaphy, *n* (%)	
Sutureless	3 (2.1)
One suture renorrhaphy	64 (45.7)
Two sutures renorrhaphy	73 (52.1)
Haemostatic agent, *n* (%)	114 (81.4)
EBL, mL, median (IQR)	150 (100–300)
Intraoperative complications, *n* (%)	2 (1.4, single‐unit blood transfusion)
Clavien–Dindo postoperative complications, *n* (%)	
I	13 (9.3)
II	9 (6.4)
IIIa	7 (5)
Pseudoaneurysm	3 (2.2)
Pneumothorax	2 (1.4)
Urinary leakage	1 (0.7)
Symptomatic lymphocele	1 (0.7)
Postoperative transfusions, *n* (%)	9 (6.4)
Length of stay, days, median (IQR)	3 (3–4)
Haemoglobin at discharge, g/L, median (IQR)	116 (102–129)
Creatinine at discharge, mmol/L, median (IQR)	0.084 (0.069–0.1052)
eGFR at discharge, mL/min/1.73 m^2^, median (IQR)	79.3 (60.2–92.5)
Discharge CKD Stage, *n* (%)	
1	40 (28.6)
2	66 (47.1)
3a	28 (20)
3b	2 (1.4)
4	4 (2.9)
5	0 (0)
Pathology, *n* (%)	
Benign	35 (25)
Malignant	105 (75)
Histological subtype, *n* (%)	
Angiomyolipoma	11 (7.9)
Oncocytoma	24 (17.1)
Clear cell RCC	67 (47.9)
Papillary RCC	22 (15.7)
Chromophobe	16 (11.4)
PSMs, *n* (%)	10 (7.1)
Pathological T Stage, *n* (%)	
1a	96 (68.6)
1b	27 (19.3)
2a	6 (4.3)
2b	2 (1.4)
3a	9 (6.4)
Follow‐up, months, median (IQR)	8 (2–11)
Creatinine at last follow‐up, mmol/L, median (IQR)	0.0875 (0.0707–0.0990)
eGFR at last follow‐up, mL/min/1.73 m^2^, median (IQR)	76 (63.4–90)
Tetrafecta achievement rate, *n* (%)	112 (80)
Intraoperative EAUiaiC complications	2 (1.4)
Postoperative Clavien–Dindo Grade ≥II complications	16 (11.4)
PSMs	10 (7.1)
eGFR decline ≥30% at 90‐day postoperative	5 (3.6)

CKD, chronic kidney disease.

### Oncological Outcomes

Histopathological analysis confirmed malignancy in 75% of cases. The predominant histology was clear cell RCC in 47.9% of cases (Table 2). Positive surgical margins (PSMs) were reported in 10 patients (7.1%). At final pathology, most patients harboured pathological (p)T1 Stage, while eight patients had pT2 disease (5.7%) and nine patients had perirenal fat infiltration (pT3a, 6.4%). At a median (IQR) follow‐up of 8 (2–11) months, no local recurrence or metastasis were observed. One patient died during the follow‐up due to metastases of colon cancer.

### Functional Outcomes, Tetrafecta Achievement, and Logistic Regression Analysis

**Fig. 2:**
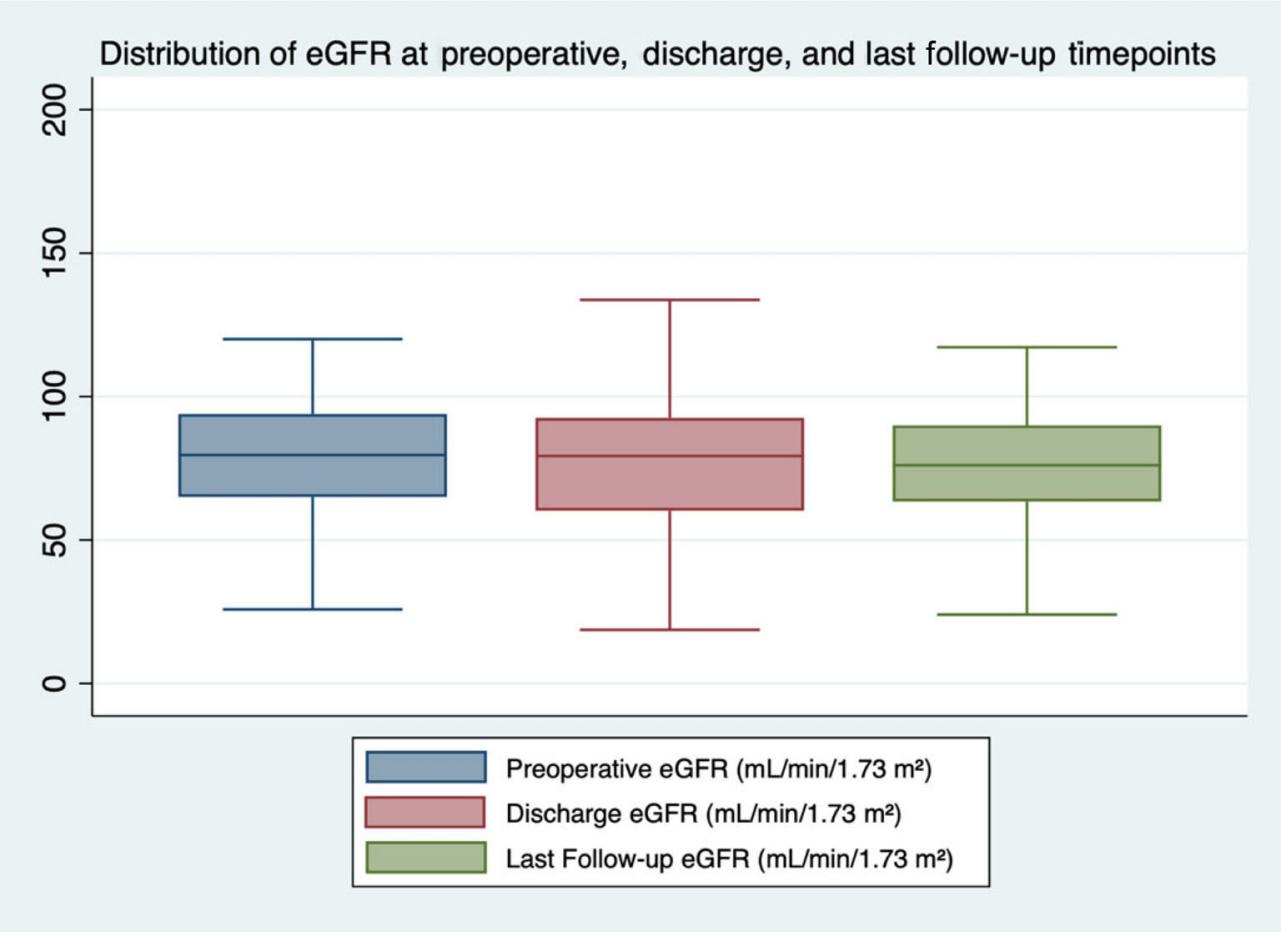
Box plots showing the distribution of eGFR measured preoperatively, at discharge, and at last follow‐up. The median is indicated by a horizontal line inside each box; whiskers represent the IQR. No statistically significant differences were observed across timepoints (repeated‐measures ANOVA, *P* = 0.244).

**Fig. 3:**
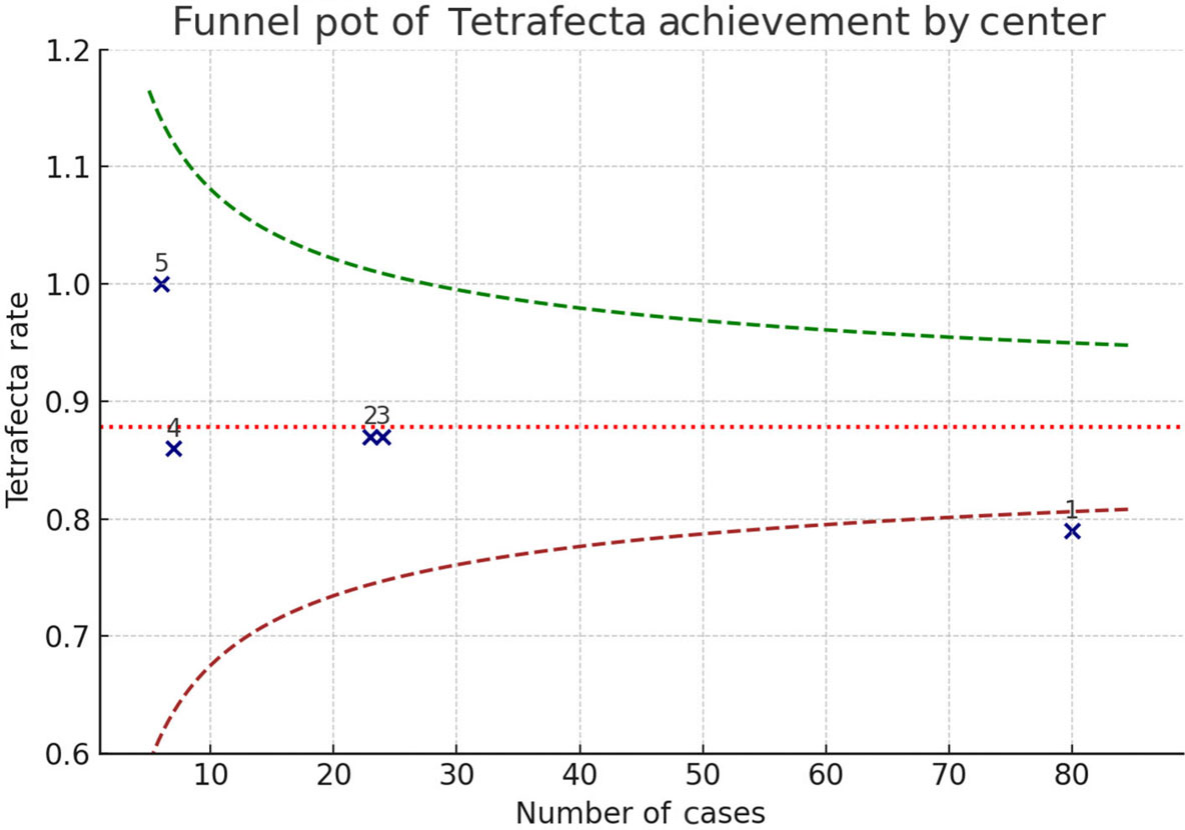
Funnel plot of Tetrafecta achievement across the five participating centres. Each dot represents one centre, plotted according to the number of procedures performed (*x*‐axis) and the observed Tetrafecta rate (*y*‐axis). The dotted line indicates the overall mean proportion, and dashed lines represent the 95% confidence limits. Centre 1 lies very close to, and marginally below, the lower confidence boundary, reflecting expected statistical variability with smaller sample sizes, which are associated with greater variability and wider confidence limits.

The median (IQR) baseline eGFR was 79.6 (65–93.9) mL/min/1.73 m^2^. At discharge, the median (IQR) eGFR was 79.3 (60.2–92.5) mL/min/1.73 m^2^. A decrease in the median (IQR) eGFR was recorded at the last follow‐up at 76 (63.4–90) mL/min/1.73 m^2^, although this was not statistically significant (Fig. 2, repeated‐measures ANOVA, *P* = 0.244). Tetrafecta was achieved in 80% of the cases (Table 2). Multivariate (adjusted) logistic regression analysis showed that the R.E.N.A.L. nephrometry score (odds ratio [OR] 0.69, 95% CI 0.52–0.93; *P* = 0.01) and Hugo RAS surgical experience (OR 1.03, 95% CI 1.01–1.06; *P* = 0.02) were independent predictors of Tetrafecta achievement (Table 3). A funnel plot analysis was conducted to evaluate inter‐centre differences in Tetrafecta achievement (Fig. 3). Four centres fell well within the 95% confidence limits, while Centre 1 was marginally below the lower limit. This finding likely reflects the greater variability associated with the smaller sample size contributed by other centres. Importantly, this deviation is small and not considered clinically meaningful, supporting overall consistency across institutions.

**Table 3:** Uni‐ and multivariate (unadjusted and adjusted) logistic regression analysis identifying predictors of Tetrafecta achievement.

Variable	Univariate (unadjusted) analysis		Multivariate (adjusted) analysis	
	OR (95% CI)	*P*	OR (95% CI)	*P*
Age	0.97 (0.93–1.01)	0.13	1.01 (0.95–1.09)	0.69
BMI	0.99 (0.91–1.08)	0.83	1.02 (0.91–1.15)	0.71
CCI score	0.85 (0.67–1.07)	0.17	0.89 (0.57–1.38)	0.60
Diabetes	2.49 (0.54–11.42)	0.24	2.64 (0.49–14.3)	0.26
Hypertension	**0.41 (0.17–0.98)**	**0.04**	0.35 (0.12–1.08)	0.07
eGFR at baseline	1.02 (0.99–1.04)	0.09	1.01 (0.98–1.05)	0.27
R.E.N.A.L. nephrometry score	**0.76 (0.61–0.96)**	**0.02**	**0.69 (0.52–0.93)**	**0.01**
Off‐ vs on‐clamp	1.87 (0.73–4.77)	0.19	1.06 (0.17–6.46)	0.95
WIT	1.06 (0.99–1.12)	0.08	1.09 (0.98–1.21)	0.12
Hugo RAS surgical experience	1.02 (0.99–1.04)	0.08	**1.03 (1.01–1.06)**	**0.02**

CCI, Charlson Comorbidity Index.

Bold values statistically significant at *P* < 0.05.

### Learning Curve Analysis

A linear regression analysis demonstrated a statistically significant inverse relationship between the progressive case number and docking time (β = −0.050, 95% CI −0.075 to −0.026; *P* < 0.001), suggesting that docking time improved with accumulated surgical experience. The model explained approximately 12.5% of the variance in docking time (*R* ^2^ = 0.125), supporting the presence of a learning curve effect (Fig. S2). Furthermore, when analysing complication rates over time, no statistically significant association was found between surgical experience and overall postoperative complications at logistic regression analysis (*P* = 0.40).

## Discussion

To date this study represents the largest multicentre case series of RAPN using the Hugo RAS system, highlighting its safety and efficacy when performed by experienced surgeons. Promising early outcomes were observed, with an 80% Tetrafecta achievement rate. Notably, no conversions to open surgery were required.

The first monocentric series of RAPN performed with the Hugo RAS system, published by Gallioli et al. [9], demonstrated encouraging results in terms of safety and feasibility. Since then, several studies on RAPN with the Hugo RAS system have been published; however, they were limited to small sample size single‐centre series, and that may have contributed to hinder the broader adoption of this platform [7, 8, 9, 10, 11].

The chance to analyse the largest cohort of RAPN procedures performed using the Hugo RAS system undoubtedly stands out as a key strength of our study. In our cohort, the median (IQR) clinical tumour size and R.E.N.A.L. nephrometry score were 32 (22–46) mm and 6 (5–8), respectively, reflecting tumours of predominantly low to intermediate complexity. Although these values are comparable to those reported in large RAPN series with the da Vinci platform, it must be acknowledged that the latter has also been widely adopted for highly complex renal masses, potentially limiting direct comparisons. Notably, our cohort demonstrated a median (IQR) console time of 79 (37–110) min and a median (IQR) EBL of 150 (100–300) mL, both of which are lower than the values reported by Veeratterapillay et al. [22] in a multicentre RAPN series (mean [SD] 141 [38] min and 205 [145] mL, respectively), even if it was a report on their early experience. Although one might expect longer operative times at the beginning of the learning curve with the Hugo RAS system—owing to the greater meticulousness and slower movements often associated with the adoption of a new platform—it is possible that the cumulative robotic experience of the urological community led to a significantly smoother and more efficient initial adoption of the Hugo RAS system compared to the early days of the da Vinci era. Moreover, the same study by Veeratterapillay et al. [22] showed a 2% rate of conversion to open/radical nephrectomy. Notably, no intraoperative major complications or conversions to open surgery occurred in our present cohort, which aligns with outcomes observed in other multicentre series, where conversion rates were <5%, even for complex tumours [23].

Compared to laparoscopic PN, a key benefit of RAPN is the ability to minimise EBL and WIT, contributing to better preservation of renal function. In our cohort, the median (IQR) WIT was 16 (12–21) min, that is significantly better than the 21 (17–27) min reported in a large Japanese multicentre study by Furukawa et al. [24], involving >800 patients, the majority of which were T1 and low‐to‐intermediate complex renal masses. This reinforces the reproducibility of RAPN outcomes across different platforms and institutional settings, particularly when treating cT1 renal tumours. Postoperative complications were graded according to the Clavien–Dindo classification [15], with 15.7% of complications classified as minor, and this was consistent with previously published data [22]. The PSM rate in our study was 7.1%, which appears slightly higher than that reported in other large series (ranging from 3.9% to 6.2%), although no formal statistical comparison was performed [25, 26]. Considering the predominantly low‐to‐intermediate tumour complexity and small median tumour size in our cohort, this finding warrants cautious interpretation and suggests potential margin for refinement in surgical technique or case selection.

In this study we proposed a novel definition of Tetrafecta which, compared to previously established metrics such as trifecta, integrates the absence of intraoperative complications. By incorporating this additional parameter, Tetrafecta may represent a more safety‐oriented metric, particularly suitable for evaluating the intra‐ and perioperative performance of emerging robotic platforms. In our cohort, we achieved a Tetrafecta rate of 80%, offering a nuanced perspective on surgical quality that accounts for both functional preservation and procedural safety. Similar frameworks, such as trifecta, have been applied in prior RAPN studies, but our Tetrafecta reflects the evolving priorities in RAPN, emphasising functional and safety outcomes in addition to oncological efficacy [27, 28, 29]. Our findings are in tune with these studies and reinforce the potential of the Hugo RAS system to meet high surgical standards in nephron‐sparing surgery (NSS). The functional outcomes observed in our multicentre cohort appear consistent with those of previous da Vinci studies [24, 27, 30], suggesting that RAPN with the Hugo RAS system may achieve high surgical standards. However, this finding should be interpreted in the context of a cohort predominantly composed of cT1 renal masses, with low‐to‐intermediate complexity, and performed by experienced surgeons.

An additional consideration emerging from this multicentre experience concerns the variability in docking configurations adopted across institutions when using the Hugo RAS system. As illustrated in Fig. 1, configurations A, B, and C—each implemented in different centres—share a similar design, with three robotic arms positioned behind the patient and a single arm placed anteriorly. This configuration facilitates a less linear trocar layout, offering greater flexibility for adapting to unconventional anatomical scenarios, such as high BMI, previous abdominal surgery, or challenging tumour locations (e.g., posterior or upper pole lesions). Conversely, configuration D, used by one centre, reflects a more linear trocar placement, similar to setups used with robotic platforms in which all robotic arms originate from a single patient cart. This standardised approach may help streamline operative setup and reduce docking time, offering the advantage of consistent workflows across different robotic systems. These findings underscore the versatility of the Hugo RAS system and its adaptability to different institutional practices. They also highlight how docking strategy can be tailored to both patient‐ and centre‐specific factors without compromising surgical outcomes.

The learning curve for RAPN, especially in complex cases, has been widely debated [17, 31, 32, 33]. However, the surgical experience with the Hugo RAS system introduces unique challenges due to its novel design, which features four robotic arms, bulkier instruments, and a mobile external unit with potentially greater risks of external collisions. These factors may influence surgical manoeuvres, including tumour enucleation. In our study the multivariate logistic regression showed that institutional experience positively influences RAPN outcomes. In fact, a better Hugo RAS surgical experience was significantly associated with increased Tetrafecta achievement (OR 1.03, 95% CI 1.01–1.06; *P* = 0.02) remarking the crucial role of surgeon familiarity with the platform in driving optimal outcomes. Similar findings were reported by Mottrie et al. [34], who observed that, despite initial variability in operative times, outcomes improved rapidly as surgeons gained familiarity with the robotic platform. Additionally, we identified the R.E.N.A.L. nephrometry score as significant predictor of Tetrafecta achievement (OR 0.69, 95% CI 0.52–0.93; *P* = 0.01), emphasising the impact of tumour complexity in influencing outcomes.

Although not a primary objective of the study, we performed two supplementary analyses to assess the learning curve. First, we evaluated docking time as a function of surgical experience, demonstrating a statistically significant reduction over time (β = −0.050, 95% CI −0.075 to −0.026; *P* < 0.001), which supports the presence of a technical learning curve related to docking optimisation. Second, we examined whether surgical experience influenced the overall complication rate through logistic regression analysis. The absence of a statistically significant association (*P* = 0.40) suggests that complication rates remained stable over time, without clear evidence of a learning‐related effect. This finding is consistent with the idea that the Hugo RAS system supports a smooth transition for surgeons, even in the early phase of adoption. The complications observed were evenly distributed and do not appear to reflect a learning curve. Instead, they are more likely attributable to patient‐related clinical variability rather than to platform‐specific challenges or surgeon inexperience. Nevertheless, given the small number of events, this conclusion should be interpreted cautiously and warrants further validation in larger cohorts.

We acknowledge that this study has some limitations, most notably its retrospective analysis, even though data were prospectively collected across participating centres. While the present study represents the largest multicentric series evaluating RAPN with the Hugo RAS system, the overall cohort of 140 patients—primarily selected for low‐to‐intermediate complexity tumours—may not fully capture the range of intra‐ and postoperative outcomes observed in high‐complexity scenarios. However, we believe it potentially reflects the heterogeneity of real‐world RAPN practice with the Hugo RAS system. Prospective studies with standardised protocols and larger sample size are needed to further validate these findings and establish clear guidelines for the optimal use of the Hugo RAS system in urological surgery. Nonetheless, the data presented here lay a solid foundation for future research, particularly regarding the evolving role of novel robotic systems in NSS. The variability in surgical techniques and perioperative management across centres may have also influenced outcomes. The low rate of on‐clamp procedures (35.7%) does not reflect any technical limitation of the Hugo RAS system. Rather, it mirrors the established surgical preferences and routines of the participating surgeons who, regardless of the robotic platform used, continued to apply their standard approach to RAPN, including their typical use (or avoidance) of clamping. This continuity of surgical behaviour suggests that transitioning from the da Vinci surgical system to the Hugo RAS system did not lead to more conservative intraoperative strategies, such as routinely opting for clamping even in cases where it might not have been necessary. In fact, one could have reasonably expected a greater tendency toward clamping when adopting a new platform, as a protective measure, possibly leading to higher clamping rates even for low‐complexity tumours. However, our data suggest the opposite: surgeons accustomed to performing off‐clamp procedures with da Vinci continued to do so with the Hugo RAS system. This observation reinforces the idea that the Hugo RAS platform enables a seamless transfer of surgical habits and techniques, supporting consistent clinical decision‐making rather than imposing adaptations due to platform‐specific constraints. We believe this further strengthens the external validity and practical relevance of our findings. A minority of cases were performed via a retroperitoneal approach, which limits insight into the platform's versatility across different surgical settings. Standardising surgical protocols would help facilitate more direct comparisons between institutions and reduce variability in outcome reporting.

Finally, although there were no specific exclusion criteria for complex renal masses, these cases were anecdotal. As such, the majority of the tumours treated were small and of low‐to‐intermediate complexity. It is understandable to assume that centres equipped with at least one additional robotic platform besides the Hugo RAS system might raise the reader's concern regarding a potential selection bias preferring the da Vinci surgical system for more complex lesions, which, within the framework of the present study design, we are unable to confirm or refute. Nonetheless, we believe this could highlight the safe applicability of the Hugo RAS system even in selected challenging NSS cases.

Despite these limitations, the present study represents the first multicentre evaluation of RAPN using the Hugo RAS system, providing a comprehensive overview of its feasibility, safety, and effectiveness across several institutions in a real‐world clinical setting. Given the diverse range of institutions and surgeons involved, our findings reflect the potential of the Hugo RAS system to perform effectively across varying levels of institutional experience and surgical techniques. However, its presumed cost‐effectiveness has yet to be formally demonstrated, as current regional tenders indicate costs comparable to those of other systems. Nevertheless, the Hugo RAS system may represent a viable option for RAPN, particularly for institutions seeking an alternative to established robotic platforms.

In conclusion, this multicentre evaluation confirms the feasibility, safety, and effectiveness of RAPN performed using the Hugo RAS system. The Tetrafecta achievement rate is satisfactory, but the risk of selection bias may be present. While further prospective studies are warranted to understand the drivers of success in RAPN better, the present study provides valuable insights into the capability of the Hugo RAS system to perform RAPN in a real‐world setting.

## Funding

None.

## Ethics Statement

The study was conducted in accordance with the Declaration of Helsinki.

## Informed Consent Statement

Informed consent was obtained from all subjects involved in the study. The patients in this manuscript have given written informed consent for publication of their data.

## Disclosure of Interests

Two of the five participating centres (including some co‐authors*) have consultancy or proctoring relationships with Medtronic (manufacturer of the Hugo™ RAS system). All other authors and centres declare no conflicts of interest. *Prof. Rocco Papalia (Fondazione Policlinico Universitario Campus Bio‐Medico). Dr Antonio Galfano (ASST Grande Ospedale Metropolitano Niguarda). Dr Paolo Dell'Oglio (ASST Grande Ospedale Metropolitano Niguarda).

## Supporting information


**Fig. S1.** Histogram showing the number of RAPN procedures performed in each participating centre.

**Fig. S2.** Scatterplot showing the docking time (min) across consecutive RAPN cases performed with the Hugo RAS system. A linear regression line is overlaid to illustrate the decreasing trend of docking time with increased surgical experience, supporting the presence of a learning curve.

**Table S1.** Intraoperative Complications Assessment and Reporting with Universal Standards (ICARUS) criteria for reporting adverse events during surgical procedures.
**Table S2.** Quality criteria for accurate and comprehensive reporting of surgical outcome to collect postoperative complications.

## Data Availability

The data presented in this study are available on request from the corresponding author.
