# Supplementary figures and images for: Robot‐assisted partial nephrectomy using the Hugo™ RAS System: first multicentre study and Tetrafecta achievement
Source: BJU Int. 2025 Oct 6;136(6):1145–55. doi: 10.1111/bju.70009 (PMC12606541; doi:10.1111/bju.70009)

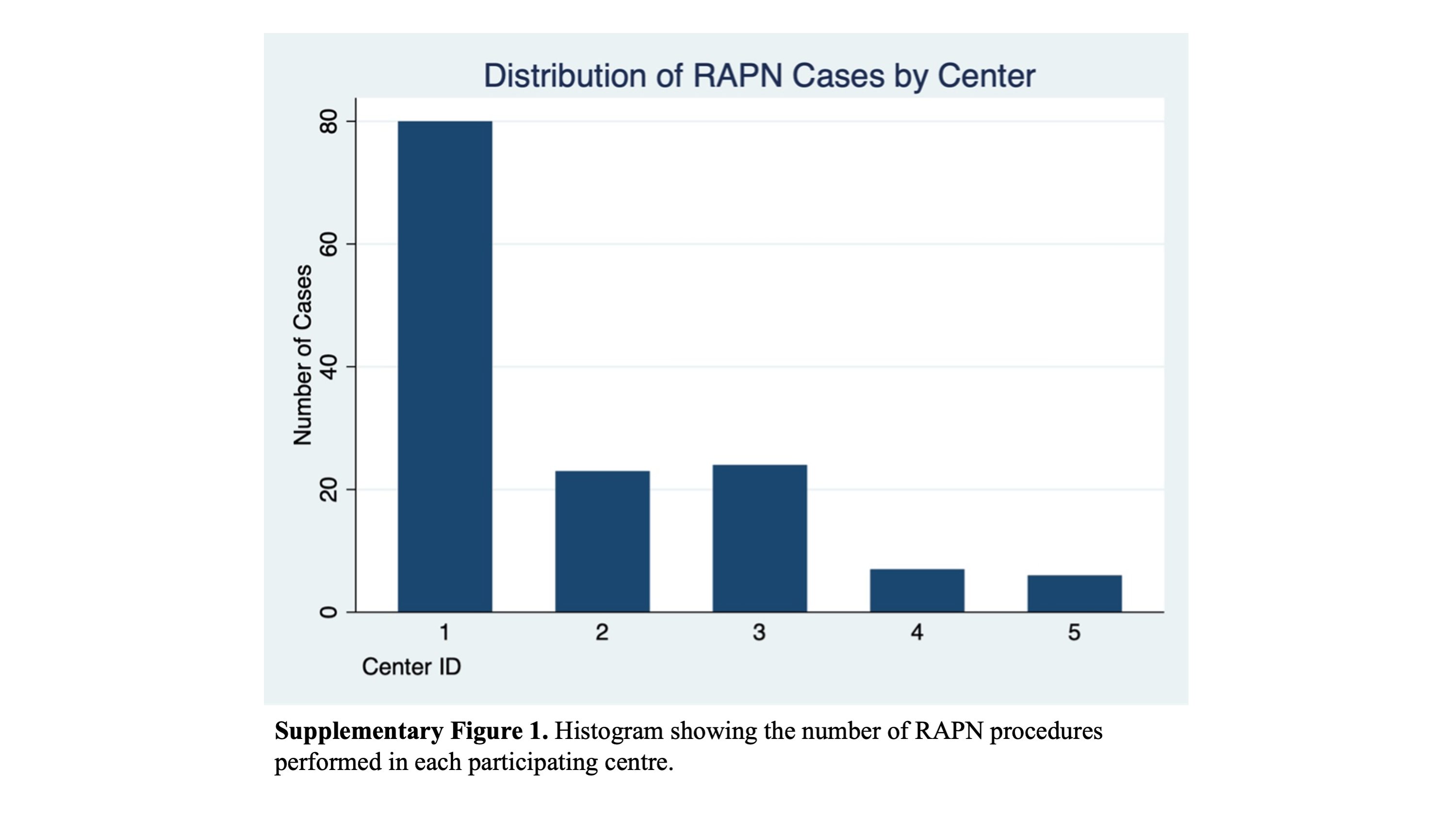

Supplement: Supplementary file 1 — Fig. S1. Histogram showing the number of RAPN procedures performed in each participating centre. [file BJU-136-1145-s003.png]

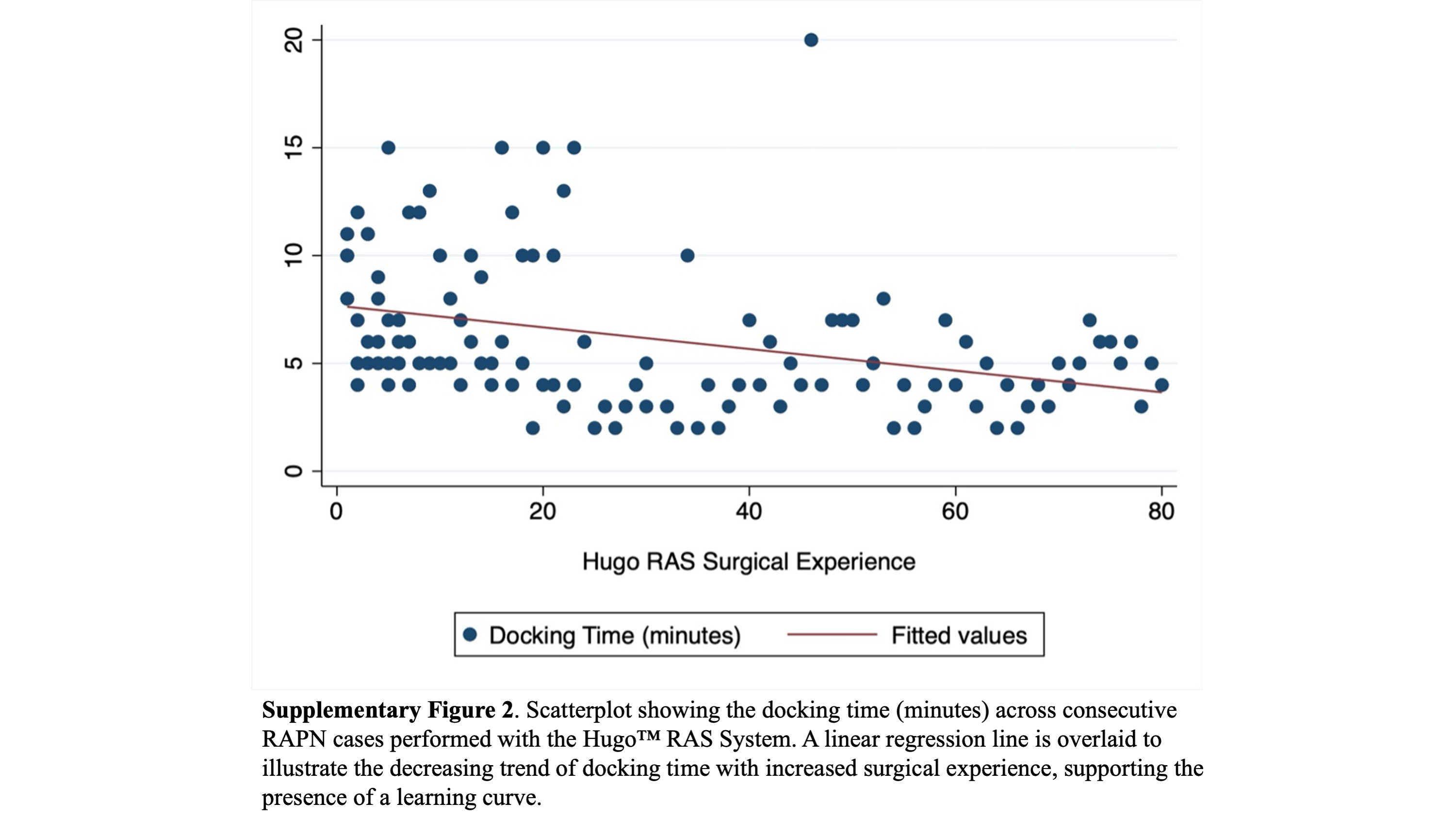

Supplement: Supplementary file 2 — Fig. S2. Scatterplot showing the docking time (min) across consecutive RAPN cases performed with the Hugo RAS system. A linear regression line is overlaid to illustrate the decreasing trend of docking time with increased surgical experience, supporting the presence of a learning curve. [file BJU-136-1145-s002.png]
