# Supplementary material for: Robot‐assisted partial nephrectomy using the Hugo™ RAS System: first multicentre study and Tetrafecta achievement
Source: BJU Int. 2025 Oct 6;136(6):1145–55. doi: 10.1111/bju.70009 (PMC12606541; doi:10.1111/bju.70009)
Supplement: Supplementary file 3 — Table S1. Intraoperative Complications Assessment and Reporting with Universal Standards (ICARUS) criteria for reporting adverse events during surgical procedures. Table S2. Quality criteria for accurate and comprehensive reporting of surgical outcome to collect postoperative complications. [file BJU-136-1145-s001.docx]

**Supplementary Table 1:** Intraoperative Complications Assessment and Reporting with Universal Standards (ICARUS) criteria for reporting adverse events during surgical procedures

| **V or X** | **Criteria** | **Explanation** |
| --- | --- | --- |
| V | 1. In a study reporting perioperative outcomes, iAEs should be reported as one of the outcomes of interest | In the current study intraoperative adverse events were one of the primary end points |
| V | 1. iAEs and the definition of each specific iAE should be reported or referenced | No iAE were recorded in the current study |
| V | 1. Each iAE should be reported using one of the proposed classification systems (ClassIntra, EAU, iAE severity classification scheme, or modified Satava), with a preference for schemes that are validated | The study was designed to categorize Intraoperative adverse events according to the Intraoperative Adverse Incident Classification (EAUiaiC) proposed by the European Association of Urology (EAU) ad hoc Complications Guidelines Panel. However, no iAE were recorded in the current study |
| V | 1. Each iAE should be reported separately by grade | No iAE was recorded in the current study. |
| V | 1. iAEs related to anesthesiology, surgery, and equipment malfunction should be reported separately | In the current study no intraoperative adverse event was associated with anesthesiology or equipment malfunctions. |
| V | 1. The number of iAEs and the number of patients with iAEs should be reported separately | No iAE was recorded in the current study |
| X | 1. When appropriate, pre-existing medical conditions, atypical anatomical variants, and malfunctioning surgical instruments associated with iAEs should be reported | No specific pre-existing medical conditions or atypical anatomical variants were available |
| V | 1. If an iAE requires conversion during surgery, both the iAE that caused the conversion and the action undertaken should be reported | In the current study no conversion to open surgery was reported |
| V | 1. iAEs should be reported, specifying the surgical step that was associated with or affected by the iAEs | No iAE was recorded in the current study |
| V | 1. The timing of iAE assessment should be reported as follows:   If an iAE is recognized during the surgical procedure, hold a debriefing after the surgical procedure. If an iAE if not recognized during the surgical procedure, report the point at which the iAE became apparent in the postoperative course | No iAE was recorded in the current study |
| V | 1. The management of iAEs should be reported | No iAE was recorded in the current study |
| V | 1. Report the clinical consequences of a given iAE in the postoperative course as follow:   a) Without postoperative sequelae  b) With nonpermanent postoperative sequelae  c) With a permanent postoperative sequela  d) Requiring reoperation  e) Postoperative death | No iAE was recorded in the current study |
| V | 1. Report changes to the clinical course that were associated with any iAEs | No iAE was recorded in the current study |

**Supplementary Table 2:** Quality criteria for accurate and comprehensive reporting of surgical outcome to collect postoperative complications

| **V or X** | **Criteria** | **Explanation** |
| --- | --- | --- |
| V | 1. Define the method of accruing data | Prospective data collection |
| V | 2. Define who collected the data | Data were collected by medical doctors who were not involved in the treatment |
| V | 3. Indicate the duration of follow-up | 30 days |
| X | 4. Include outpatient information | Outpatient information were not collected |
| X | 5. Include mortality data and causes of death | Mortality data and cause of death were not collected |
| V | 6. Include definitions of complications | Complications were defined as any deviation from the ideal postoperative course |
| V | 7. Define procedure-specific complications | Complications directly related to surgery were specifically reported. |
| V | 8. Report intraoperative and postoperative complications separately | Intraoperative and postoperative complications were reported separately |
| V | 9. Use a severity grading system for postoperative complications | The Clavien-Dindo system was used |
| V | 10. Postoperative complications should be presented in a table either by grade or by complication type | Postoperative complications were presented in a table by both complication type and Clavien-Dindo grade. |
| V | 11. Include risk factors | The CCI and ASA score were prospectively collected for all patients. |
| V | 12. Include readmissions and causes | Four readmissions (2.9%) were recorded during the study period. |
| V | 13. Include reoperations, types and causes | No reoperation was recorded during the study period. |
| V | 14. Include the percentage of patients lost to follow-up | No patient was lost to follow-up. |
| Legend: CCI = Charlson Comorbidity-index; ASA = American Society of Anesthesiologists score | | |
